# Recovering superhydrophobicity in nanoscale and macroscale surface textures†

**DOI:** 10.1039/c9sm01049a

**Published:** 2019-08-20

**Authors:** Alberto Giacomello, Lothar Schimmele, Siegfried Dietrich, Mykola Tasinkevych

**Affiliations:** Sapienza Università di Roma, Dipartimento di Ingegneria Meccanica e Aerospaziale 00184 Rome Italy alberto.giacomello@uniroma1.it +39 06 44585200; Max-Planck-Institut für Intelligente Systeme 70569 Stuttgart Germany; IV. Institut für Theoretische Physik, Universität Stuttgart 70569 Stuttgart Germany; Centro de Física Teórica e Computacional, Departamento de Física, Faculdade de Ciências, Universidade de Lisboa Campo Grande P-1749-016 Lisboa Portugal

## Abstract

Here, we investigate the complete drying of hydrophobic cavities in order to elucidate the dependence of drying on the size, the geometry, and the degree of hydrophobicity of the confinement. Two complementary theoretical approaches are adopted: a macroscopic one based on classical capillarity and a microscopic classical density functional theory. This combination allows us to pinpoint unique drying mechanisms at the nanoscale and to clearly differentiate them from the mechanisms operational at the macroscale. Nanoscale hydrophobic cavities allow the thermodynamic destabilization of the confined liquid phase over an unexpectedly broad range of conditions, including pressures as large as 10 MPa and contact angles close to 90°. On the other hand, for cavities on the micron scale, such destabilization occurs only for much larger contact angles and close to liquid–vapor coexistence. These scale-dependent drying mechanisms are used to propose design criteria for hierarchical superhydrophobic surfaces capable of spontaneous self-recovery over a broad range of operating conditions. In particular, we detail the requirements under which it is possible to realize perpetual superhydrophobicity at positive pressures on surfaces with micron-sized textures by exploiting drying, facilitated by nanoscale coatings. Concerning the issue of superhydrophobicity, these findings indicate a promising direction both for surface fabrication and for the experimental characterization of perpetual surperhydrophobicity. From a more basic perspective, the present results have an echo on a wealth of biological problems in which hydrophobic confinement induces drying, such as in protein folding, molecular recognition, and hydrophobic gating.

## Abstract 2

We investigate drying of hydrophobic cavities and its dependence on the size, the geometry, and the hydrophobicity of the confinement. The emerging scheme of drying from the nano- to the macroscale allows one to understand perpetual superhydrophobicity.

## Graphical abstract

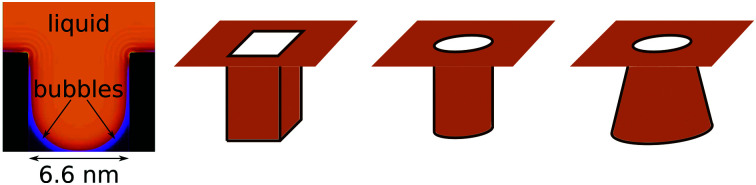

## 1 Introduction

Several interfacial phenomena of technological interest run under the name of superhydrophobicity: self-cleaning,^1^ anti-fouling,^2^ drag reduction,^3^*etc.* Such behaviors are tied to the occurrence of a “suspended”, so-called Cassie–Baxter, state in which a liquid body is in contact not only with a solid surface but also with vapor or gas pockets which occupy surface asperities, sustained by capillary forces. However, this superhydrophobic state is fragile, in the sense that external factors, such as pressure variations, contaminants, or mechanical vibrations, can cause its collapse into the Wenzel state in which all surface asperities are wet.

If present, perpetual superhydrophobicity promises to heal the fragility of a suspended superhydrophobic state, thus opening novel scenarios for applications in materials science. It has been shown^4^ that the Wenzel state can become thermodynamically unstable in nanoscale hydrophobic cavities, making the superhydrophobic state capable of self-recovery when the pressure is lowered to ambient conditions. However, direct experimental evidence of perpetual superhydrophobicity is still lacking for surfaces close to application interests, due to conceptual, experimental, and fabrication difficulties. Here, we clarify the concept of perpetual superhydrophobicity in its various connotations at the macro and nanoscale, with the goal of proposing new design criteria for superhydrophobic surfaces and stimulating accurate experimental studies of this drying phenomenon. Drying is an interfacial phase transition in which, upon changing pressure or temperature, the solid–liquid interface is replaced by a solid–vapor and vapor–liquid interface.^5^

Understanding drying in hydrophobic confinement is crucial not only for superhydrophobicity but also for a number of important biological phenomena. For proteins (or large molecules) in solution the proximity of hydrophobic residues can give rise to local evaporation and, as a consequence, to hydrophobic attraction.^6,7^ This mechanism is known to play a role in protein folding,^8,9^ molecular recognition,^10^ self-assembly,^11^ and hydrophobic gating^12,13^ – to name a few systems. The results discussed here, with a particular focus on superhydrophobic surfaces, are of relevance for this panoply of phenomena, in the sense that they contribute to disentangle the complex dependence of drying on the size, the geometry, and the degree of hydrophobicity of the confinement. In particular, we investigate four textures: two-dimensional grooves, cylindrical, parallelepipedic, and ink-bottle-shaped pits which are representative of different degrees of confinement and of relevant geometrical characteristics, such as corners, tapered walls, *etc.* In principle, currently available techniques can be used for producing nanotextured surfaces with similar geometries: quasi-2D grooves, cylindrical pits, and tapered cylinders.^14^

Superhydrophobicity originates from capillary forces which are capable of sustaining a liquid–gas interface atop rough surfaces – the so-called Cassie–Baxter state. However, in a typical situation, pressure variations or other changes of the environmental conditions may overcome the capillary forces and induce complete wetting of the rough surface (Wenzel state), with the concomitant loss of superhydrophobic properties. The reverse transition from the Wenzel state to the Cassie–Baxter state is usually characterized by large free-energy barriers^15–17^ which prevent the recovery of superhydrophobicity under ambient conditions without an external supply of free energy. Various strategies for active recovery have been proposed including the application of electric^18–22^ or magnetic fields,^23^ illumination by ultraviolet light,^24,25^ or heating of the surface.^26,27^ On the other hand, self-recovery aims at destabilizing the Wenzel state by purely passive means in order to completely and economically realize perpetual superhydrophobicity, at least over a range of technologically relevant pressures.

There have been several attempts to realize “reversible”^28^ or “monostable”^29^ superhydrophobicity; for highly non-wetting liquids such as mercury, perpetual superhydrophobicity was reported for surfaces decorated with micron-scale textures.^29^ For water, instead, due to its higher affinity for numerous substrates (lower hydrophobicity), this simple approach is not viable and the breakdown of superhydrophobicity proves irreversible even when the textures are as small as 20 nm.^30^ Therefore, surfaces with hierarchical roughness, one on the micron and one on the nanometer scale, were proposed.^28,29^ This promising strategy relies on the intuition that the smaller tier of roughness remains always dry due to nanoconfinement-enhanced hydrophobicity and promotes the drying of the larger tier. However, ensuring that nanoscale roughness is perpetually dry, and that the larger textures inherit this property, is highly nontrivial^31^ and is experimentally arduous to verify. The present study aims at providing a coherent picture of drying from the nano- to the macroscale and formulating the conditions under which these mechanisms can be combined to realize self-recovering hierarchical surfaces, providing the hitherto missing theoretical tools to understand and design perpetual superhydrophobicity. In the following, we consider that there is no gas dissolved in water but only its vapor phase is formed. The degassed liquid represents the most unfavorable condition for the survival of superhydrophobicity.^32,33^

To the best of our knowledge, spontaneous recovery of superhydrophobicity has not been demonstrated unequivocally in experiments with water. The main experimental difficulties are that (i) it is challenging to realize and to characterize regular surface textures on the nanometer scale, and (ii) the pressures required in order to press the liquid into such textures – in order to successively observe its spontaneous retraction – are exceptionally high. However, for porous materials high-pressure intrusion and extrusion experiments have shown that spontaneous drying can indeed be achieved in hydrophobic nanopores.^34,35^ Although these systems, composed of nanoporous granules immersed in water, are quite distinct from superhydrophobic surfaces concerning shape and technological applicability, they positively demonstrate the physical mechanism of spontaneous drying at the nanoscale (based on which perpetual superhydrophobicity can be realized): after liquid intrusion into the pores at high pressures, it is possible to trigger their drying simply by decreasing the pressure below a certain threshold value, which we call the drying pressure *P*_dry_. Thus, for the smallest pores, around 2 nm wide or below, drying was observed even at pressures as high as tens of megapascals.^35–37^ This phenomenon is related to the thermodynamic destabilization of the liquid phase due to nanoconfinement, which may occur even under “normal” conditions; this fact could also be exploited for designing materials endowed with perpetual superhydrophobicity. Importantly, the drying pressure is controlled by the wetting and the geometric characteristics of the pores, a topic which is rationalized in the present study and which is crucial for designing surfaces featuring perpetual superhydrophobicity.

On the theoretical side, early microscopic simulations have shown^38–40^ that free-energy barriers for drying decrease with increasing confinement; more recent simulation work has demonstrated barrierless drying in nanostructured hydrophobic surfaces with various geometries.^41,42^ Microscopic (classical) density functional theory (DFT) calculations have also confirmed barrierless drying in nanoscale grooves^4^ with moderately hydrophobic surfaces (*i.e.*, contact angles *θ*_Y_ slightly above 90°). Finally, macroscopic theories predict that it is possible to realize drying at pressures which are much higher than the liquid–vapor coexistence pressure *P*_coex_(*T*) (at a given temperature *T*) even for large-scale textures, provided that the surfaces are sufficiently hydrophobic, with the contact angle *θ*_Y_ being typically much larger than in the nanoscale case.^32,42^ Viewed together, these studies underscore an important conclusion: drying is very sensitive to geometry, hydrophobicity, and roughness size, which are the main design parameters for perpetual superhydrophobicity. Another important aspect, which has not yet been fully appreciated, is that the drying mechanism at the macroscopic scale, which is well-known in the special case of a wedge,^43–46^ apparently differs from and seems to be unrelated to the nanoscale one which favors drying in a much broader range of thermodynamic conditions and material parameters.^4^

In view of the paramount technological interest, the scatter of theoretical results, and the difficulty in realizing and interpreting experiments, it is urgent to clarify the physical mechanisms of drying as well as to uncover their dependence on material parameters with particular attention to size-dependence. Here, we address this issue by devising a generalized macroscopic theory of drying in confinement (Section 2), by studying *via* microscopic DFT drying in a variety of geometries (Section 3), and by discussing drying on surfaces with multiple roughness scales (Section 4). The coherent picture of drying, which emerges from this multiscale analysis, provides crucial guidelines for designing perpetual superhydrophobicity and for clearly interpreting corresponding experiments on (perpetual) superhydrophobicity.

## 2 Macroscopic drying mechanism

In this section, we recall the existing macroscopic theory of drying in infinitely extended corners (“wedge drying”) and develop the missing macroscopic formulation for simple textures of finite size and at generic pressures. The macroscopic drying mechanism is expected to be relevant for surface textures being larger than a few tens of nanometer and to be suitable only at large contact angles *θ*_Y_. Additionally, the interval of pressures Δ*P*_dry_ (above and below bulk liquid–vapor coexistence), for which drying occurs, rapidly reduces as the characteristic dimension *w* of such textures increases; as will be shown below, one finds the scaling behavior Δ*P*_dry_ ∼ 1/*w*.

The wetting properties of the solid walls forming the wedge are characterized by Young's contact angle cos *θ*_Y_ ≡ (*γ*_sv_ − *γ*_sl_)/*γ*_lv_, where *γ*_sv_, *γ*_sl_, and *γ*_lv_ are the surface tensions of the solid–vapor, solid–liquid, and liquid–vapor interfaces, respectively. In order to find the value *θ*_wd_ of *θ*_Y_ above which an infinitely extended wedge dries at bulk liquid–vapor coexistence, the following classical argument is considered, inferred from ref. 43 by exchanging liquid and vapor therein (see also references therein, *e.g.*, ref. 47). An infinitely extended wedge – a geometry formed by two half planes meeting at a given angle *ψ* – is filled with liquid, except for a vapor filled region, *i.e.*, a vapor bubble, occupying the wedge corner. At bulk liquid–vapor coexistence the pressures in the liquid (*P*_l_) and in the vapor phase (*P*_v_) are equal and given by the coexistence pressure *P*_coex_(*T*), *P*_l_ = *P*_v_ = *P*_coex_(*T*). Under these conditions, mechanical equilibrium requires a planar liquid–vapor interface meeting the walls of the wedge precisely at the contact angle *θ*_Y_ characterizing the walls. This construction is only possible for a specific value *θ*_wd_ of the contact angle at which the liquid–vapor interface is in an indifferent equilibrium, *i.e.*, its translation does not cause any change of free energy. For a wedge with an opening angle *ψ*, this condition reads1
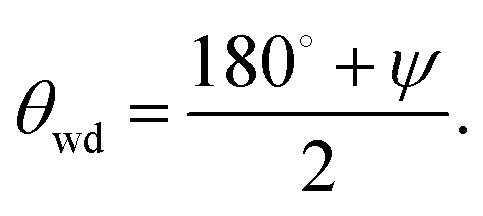
For a rectangular wedge one has *θ*_wd_ = 135°. For *θ*_Y_ < *θ*_wd_ the liquid–vapor interface in the wedge is unstable and the vapor bubble shrinks to zero; for *θ*_Y_ > *θ*_wd_ the position of the liquid–vapor interface becomes unstable and it moves to infinity, drying the wedge.

Microscopic DFT studies of wedge drying or of the related phenomenon of wedge filling clearly show a vapor-filled region at the wedge corner of hydrophobic walls or a liquid region in the wedge filled by vapor in the case of hydrophilic walls.^43–46^

It is easy to extend the purely geometric construction leading to eqn (1) to a three-dimensional corner formed by three orthogonal planes:2
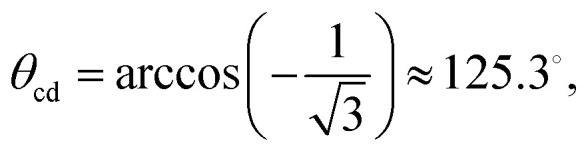
where the subscript “cd” stands for “corner drying”.

To the best of our knowledge, there have been only limited attempts to extend the concept of wedge drying to geometries in which the maximum size of a vapor bubble in the geometry is restricted due to the presence of further confining walls. We are also not aware of attempts to explore the consequences of such conditions off-coexistence, *i.e.*, for Δ*P* ≡ *P*_l_ − *P*_coex_ ≈ *P*_l_ − *P*_v_ ≠ 0. Here, we analyze, within the macroscopic framework of classical capillarity, the conditions for drying in rectangular grooves, focusing on the effects due to the finite width of the grooves. The adaptation of wedge drying theory to include these geometrical constraints provides an understanding of the limit of stability for the Wenzel state in actual, finite-size textures, enabling the determination of the pressure difference Δ*P*_dry_ below which the groove remains unconditionally dry, *i.e.*, the confined liquid phase becomes thermodynamically unstable.

The first step to formulate a macroscopic theory of drying in rectangular grooves is to express the grand canonical potential within the classical-capillarity approximation:^48,49^

3 *Ω* = Δ*PV*_v_ + *γ*_lv_(*A*_lv_ + cos *θ*_Y_*A*_sv_),

where *V*_v_ is the volume of the vapor bubble, *A*_lv_ is the area of the (sharp) liquid–vapor interface, and *A*_sv_ is the area of the solid–vapor interface, and Young's law for cos *θ*_Y_ has been used which relates the surface tensions and the contact angle. The fully wet state, for which *V*_v_ = 0, *A*_lv_ = *A*_sv_ = 0, and *Ω* = 0, is chosen as the reference state. Eqn (3) is just a sum of bulk and surface terms; the sign of the first one depends on Δ*P*, the second is always positive, while the third one is negative for hydrophobic substrates (*θ*_Y_ > 90°).

In the second step, we follow an extension of the classical nucleation theory to confined geometries^17,50^ in order to calculate the profile *Ω*(*V*_v_) of the constrained grand potential related to the nucleation of vapor bubbles. Accordingly, *Ω*(*V*_v_) is obtained by minimization of eqn (3), constrained to a given value of the order parameter *V*_v_. This approach yields the sequence of the most probable bubble configurations and provides access to stable and metastable states – corresponding to global and local minima of the grand potential, respectively – and to the grand-potential barriers separating them. Importantly for the present study, it is possible to identify the spinodal conditions for which the grand-potential barrier related to drying vanishes, which renders the sought drying pressure Δ*P*_dry_.

The results of this procedure are illustrated in Fig. S1 (ESI†) for infinitely deep grooves with *θ*_Y_ = 145°. The grand-potential profile close to *V*_v_ = 0 corresponds to two identical vapor bubbles occupying the two corners at the bottom of the groove; the curvature of the liquid–vapor interface decreases as *V*_v_ increases. We remark that the “Wenzel” minimum actually does not occur for a fully wet groove (*V*_v_ = 0), as instead is the case for contact angles *θ*_Y_ < *θ*_wd_,^17,32^ but for two finite bubbles occupying the corners of the groove. The volume of each of these stable bubbles grows as Δ*P* decreases from positive values towards bulk coexistence. At *V*_v_ = *V*_2b_ (marked by a star in Fig. S1, ESI†) the two bubbles touch each other. This condition is used to locate the groove drying pressure Δ*P*_dry_. Indeed, for Δ*P* infinitesimally below Δ*P*_dry_, the topology of the liquid–vapor interface changes, with the two disconnected pieces of interface merging into one spanning the entire groove. The position of this merged interface could only be stabilized at the much higher coexistence pressure of capillary vapor and capillary liquid. Therefore, the merged liquid–vapor interface detaches from the bottom of the groove and slides without a barrier up to the mouth of the groove, completely drying it (Fig. S2, ESI†). Clearly, this drying mechanism is related to the finite width of the groove which permits the merging of the two corner bubbles. In the case that both the width and the depth of the groove are “infinitely” large, it is possible, instead, to accommodate (meta)stable corner bubbles of any size (thin lines in Fig. S1, ESI†), excluding the occurrence of groove drying for Δ*P* > 0 due to Δ*P*_dry_ → 0 for *w* → ∞.

It is interesting to note that for grooves with *θ*_Y_ < *θ*_wd_, a case considered in ref. 17 and 32, the macroscopic theory always predicts a finite barrier for drying, even for Δ*P* < 0. In other words, the macroscopic expectation is that there is no spinodal drying, *i.e.*, no barrierless drying in grooves for *θ*_Y_ < 135°, whatever the pressure. As it will be shown in the next section, this result is a direct consequence of the classical-capillarity approximation. In addition, the present macroscopic theory neglects thermal fluctuations, which can easily overcome barriers of the order of *k*_B_*T*, where *k*_B_ is the Boltzmann constant and *T* the temperature, leading to a kinetic criterion for drying (see, *e.g.*, ref. 42 and 51).

A further comment is in order concerning the typical angle *θ*_Y_ at which drying occurs, *e.g.*, 135° for rectangular grooves. This value is unrealistically large for the contact angle of water on a smooth hydrophobic surface. One should however consider first that there are other liquids which exhibit such large values of the contact angle, *e.g.*, mercury,^29^ to which the present results apply. Secondly, geometries different from the rectangular wedge (see, *e.g.*, eqn (2)) and nanoscale confinement (see Fig. 2) can significantly decrease the drying contact angle. Thirdly, in Section 4, hierarchical textures are proposed which allow one to overcome the practical limitation imposed by the typical angle *θ*_Y_ of water.

**Fig. 1:**
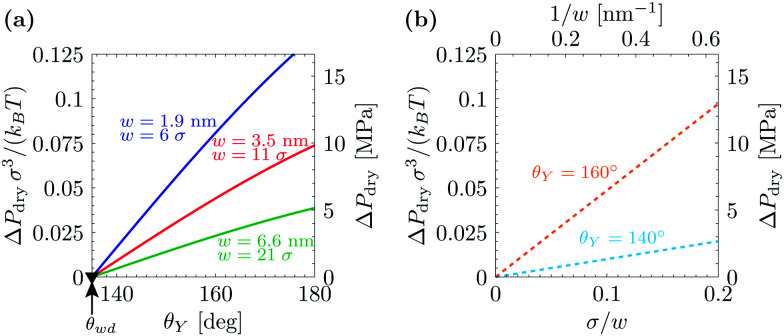
(a) Drying pressure for grooves of width *w* as a function of Young's contact angle *θ*_Y_, according to the macroscopic prediction. Connection with the DFT calculations below is established by using in the macroscopic theory the relevant values for *γ*_lv_ and *w*: *γ*_lv_ = 0.406 × *k*_B_*T*/*σ*; *w*/*σ* = 6, 11, and 21, where *σ* is the diameter of the fluid particles. The second *y* axis is in actual units, obtained by using *σ* = 0.315 nm and *k*_B_*T* = 4.16 × 10^−21^ J. The triangle at *θ*_wd_ = 135° is the wedge drying condition (1) attained at bulk liquid–vapor coexistence (Δ*P* = 0). (b) Drying pressure as a function of the inverse groove width 1/*w*, which turns out to be linear.

Fig. 1 summarizes the predictions of the macroscopic theory for drying of grooves of varying width and contact angle. Both DFT units (the fluid particle diameter *σ* and the energy unit *k*_B_*T*) and actual units are used, obtained by assuming *σ* = 0.315 nm and *k*_B_*T* = 4.16 × 10^−21^ J. The first important finding, which differs from the simple wedge drying equation (eqn (1)), is that, for *θ*_Y_ > *θ*_wd_, the finite width of the groove allows for drying at Δ*P* > 0. This confinement effect is enhanced in smaller cavities and is particularly effective at the nanoscale, where drying is expected even at pressures as large as 10 MPa for grooves with a width of 2 nm; this estimate is obtained from using *θ*_Y_ = 160°, *σ* = 0.315 nm, and *k*_B_*T* = 4.16 × 10^−21^ J. (The limitations of such macroscopic models at the nanoscale will be discussed in Section 3.) As the width of the groove is increased, Δ*P*_dry_ rapidly decreases ∝1/*w* (see Fig. 1(b)). For instance, using *θ*_Y_ = 160°, *σ* = 0.315 nm, *k*_B_*T* = 4.16 × 10^−21^ J, and *w* = 200 nm, the macroscopic prediction is Δ*P*_dry_ ≈ 1 atm. This result implies that for hydrophobic cavities of a characteristic size of *ca.* 1 µm completely dry cavities are observed in practice only at bulk coexistence and only for *θ*_Y_ ≥ *θ*_wd_. Δ*P*_dry_ increases approximately linearly with *θ*_Y_, and Δ*P*_dry_(*θ*_Y_ = *θ*_wd_) = 0. The second important conclusion is that, for *θ*_Y_ < *θ*_wd_, the grooves never undergo spinodal drying, *i.e.*, the formation of vapor bubbles in the groove is always associated with a free-energy barrier;^17,32^ therefore, in such conditions, perpetual superhydrophobicity is not possible according to the macroscopic theory of capillarity.

## 3 Microscopic drying mechanism

The macroscopic theory predicts that for *θ*_Y_ > *θ*_wd_ nanoscale hydrophobic confinement can induce drying at pressures much larger than those at bulk liquid–vapor coexistence (Fig. 1; Δ*P*_dry_(*θ*_Y_ > *θ*_wd_) > 0), *e.g.*, Δ*P*_dry_ = 10 MPa for grooves with a width of 2 nm; this estimate is obtained from using *θ*_Y_ = 160°, *σ* = 0.315 nm, and *k*_B_*T* = 4.16 × 10^−21^ J. At these scales, however, the macroscopic assumptions may turn out to be unreliable. In this section we investigate nanoscale effects on drying by performing density functional theory (DFT)^52–54^ calculations, which capture the actual nonzero widths of diffuse interfaces as well as microscopic effects in confined fluids, such as layering at the walls and specific density inhomogeneities at the corners. For details concerning the DFT calculations, the reader is referred to the Appendices, to the ESI†, and to previous studies.^4,55^ In addition, we examine the effect of the geometry of nanoconfinement by considering cavities of different shape and size, *i.e.*, quasi-two-dimensional grooves and cylindrical, parallelepipedic, and ink-bottle-shaped pits (see the ESI† for details).

**Fig. 2:**
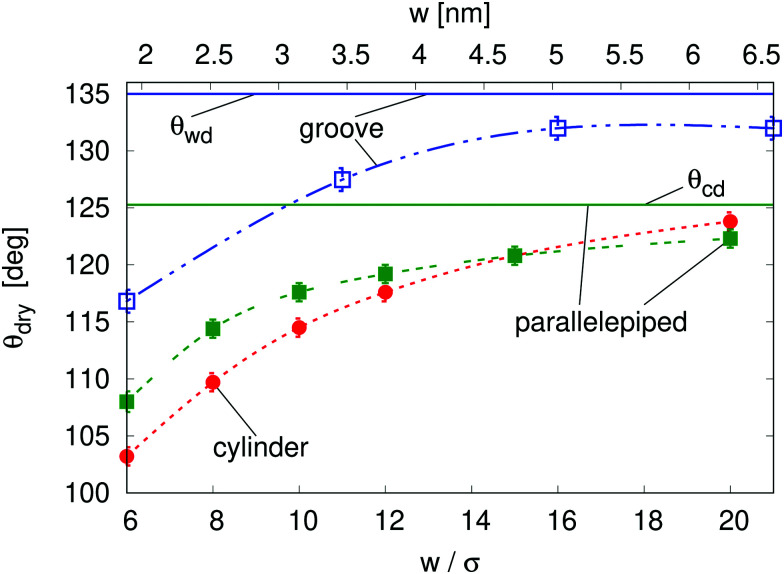
Drying angle *θ*_dry_ at Δ*P* = 0 as a function of the cavity dimension computed by microscopic DFT calculations for cylindrical (red circles) and parallelepipedic (green filled squares) pits and for the groove (blue empty squares); the lines are guides to the eye. Error bars are computed as half the difference between the values of *θ*_Y_ before and after drying. The second *x* axis is in actual units, obtained by using *σ* = 0.315 nm. The macroscopic predictions are reported for comparison for those cases in which an analytical expression is available: *θ*_wd_ = 135° for the groove and *θ*_cd_ = 125.3° for the parallelepipedic pit. As the trend indicates, it is expected that, in the limit *w* → ∞, *θ*_dry_ tends to the macroscopic predictions *θ*_wd_ for the groove and *θ*_cd_ for the parallelepipedic pit. The trend indicates that these limits are attained rather slowly.

One important nanoscale effect becomes apparent upon observing how the contact angle *θ*_dry_, at which drying occurs at liquid–vapor coexistence (Δ*P* = 0), depends on the size of the cavity (Fig. 2). On macroscopic grounds no such dependence is expected, as exemplified by the wedge and corner drying predictions in eqn (1) and (2), respectively. The reason is that the solutions to the equations of classical capillarity are self-similar, meaning that the geometry of macroscopic bubbles does not depend on their absolute size. In contrast, at the nanoscale, the size of the liquid and wall particles as well as the range of the fluid–fluid and fluid–wall interactions introduce intrinsic length scales to the problem, which influence the effective hydrophobicity of the cavity, the bubble shape, and its interaction with the neighboring bubble. In general, smaller cavities tend to facilitate drying, decreasing *θ*_dry_ by more than 15° as compared to the macroscopic expectation (see below the comparison of the macroscopic expectations with the DFT results for the parallelepipedic pit and the groove of size *w* = 6*σ* in Fig. 2). This effect is reduced as the size of the cavity increases, tending towards the macroscopic limit (*θ*_wd_ for the groove and *θ*_cd_ for the parallelepipedic pit). In other words, nano-confinement pushes the boundaries of the drying transition deeper into the range of only moderately hydrophobic materials, for which the macroscopic theory would exclude the possibility of achieving perpetual superhydrophobicity.

**Fig. 3:**
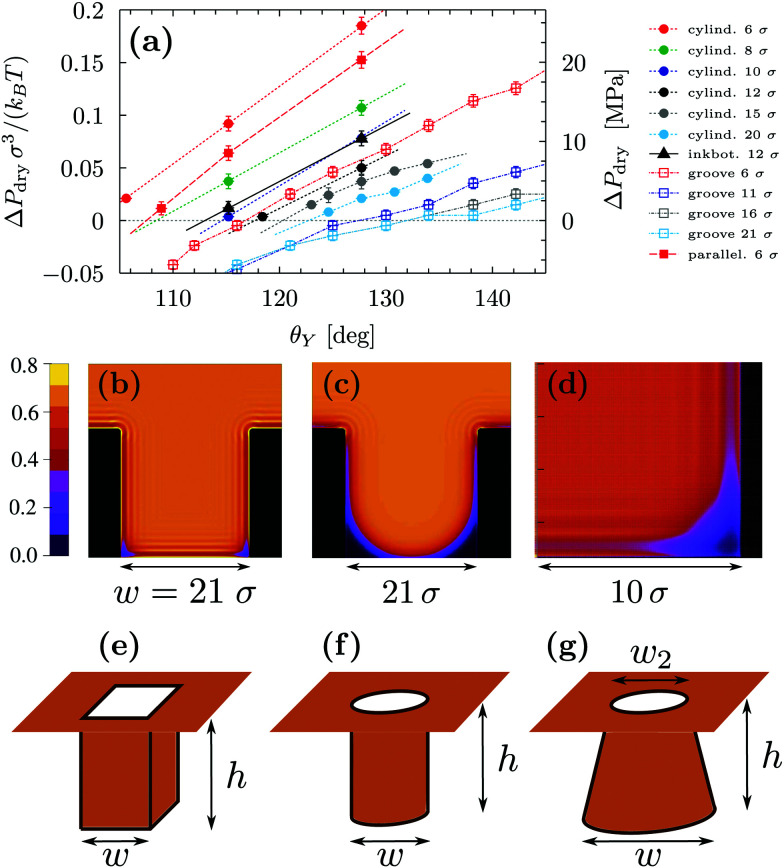
(a) Drying pressure as a function of the substrate contact angle computed by microscopic DFT calculations for four confining geometries, sketched in panels (b) and (e–g). The characteristic dimension *w* of the cavities (side legend) is taken to be the width of the two-dimensional groove (b), the side of the square base for parallelepipedic pits (e), the base diameter for cylindrical pits (f), and the diameter of the lower base for the ink bottle (g). The ink bottle is a right, circularly truncated cone with an upper diameter of 6*σ* and with a base diameter of 12*σ*. The hard-sphere diameter *σ* and *k*_B_*T* serve as the units of length and energy, respectively. The second *y* axis is in actual units, obtained by using *σ* = 0.315 nm and *k*_B_*T* = 4.16 × 10^−21^ J; for visual clarity, in the legend actual units are not reported explicitly. Due to higher computational costs, for the pits the calculations have been restricted to *θ*_Y_ > *θ*_dry_ (corresponding to Δ*P*_dry_ > 0) and to smaller *θ*_Y_ intervals than explored for the grooves; this range is that of foremost practical interest. (b) Number density distributions for the groove with width *w* = 21*σ* and *θ*_Y_ = 121°. (c) Number density distributions for the groove with width *w* = 21*σ* and *θ*_Y_ = 161°. These two configurations correspond to the “Wenzel” state with Δ*P* slightly above the corresponding drying pressure Δ*P*_dry_. (d) Number density distribution for a cylindrical pit with diameter *w* = 20*σ* and *θ*_Y_ ≈ 131°; Δ*P* is set slightly above the corresponding drying pressure Δ*P*_dry_. The sidebar provides the color code for the number density in (b–d).

In more detail, the size-dependent shift of the drying contact angle *θ*_dry_(*w*) at coexistence to values considerably smaller than the macroscopic expectation *θ*_wd_ might be understood as follows. Hydrophobic walls close to a corner are effectively more hydrophobic than the infinitely extended planar wall for which the contact angle *θ*_Y_ is defined. The disturbed balance between the fluid–fluid and the wall–fluid interactions at corners as well as the mentioned interference of wall-induced density oscillations leads to this change of the wetting properties. The enhanced hydrophobicity at corners promotes the growth of corner bubbles beyond the macroscopic estimate. Small corner bubbles are much more influenced by the locally enhanced hydrophobicity than big ones, because far away from corners the aforementioned effects are reduced. Therefore, the macroscopic condition for drying, *i.e.*, that the corner bubbles have to touch in the center of the pit or of the groove, implies that *θ*_dry_(*w*) for very wide pits or grooves, *i.e.*, large touching corner bubbles, almost coincides with the macroscopic expectations. In contrast, for very narrow pits or grooves, *i.e.*, small touching corner bubbles, a shift to much smaller values of *θ*_dry_ is observed. Other nanoscale effects, which point in the same direction, are the nonzero width of the vapor–liquid interface, the density oscillations at the pit or groove bottom, and the complex intrinsic structure of the vapor–liquid interface in nano-confinement (see Fig. 3(d)). In the presence of these effects the bubble-contact condition for drying becomes fuzzy and drying of a nanocavity may already occur for bubbles which are smaller than implied by the macroscopic condition. These nanoscale effects are in principle relevant also to wider pits or grooves; however, their relative importance decreases with increasing width. All the nanoscale effects discussed above are also relevant to the determination of the drying pressure Δ*P*_dry_ discussed below.

Fig. 3(a) reports Δ*P*_dry_ computed *via* three-dimensional DFT for four geometries. This shows that, for the same characteristic dimension, the shape of the confinement has a major effect on drying, with the following order of the cavities with the most destabilizing effect on the liquid: ink-bottle, cylindrical pit, parallelepipedic pit, groove. This trend reflects the fact that increasing the number of hydrophobic confining surfaces, *e.g.*, switching from a two-dimensional groove to a three-dimensional pit, facilitates drying. Similarly, the presence of acute corners promotes drying, which is revealed by comparing cylinders with ink bottles. Comparing the drying behavior of cylinders and parallelepipeds is more subtle, because for the smallest ones (6*σ*) the cylindrical geometry encloses a smaller volume of liquid, thus favoring drying (compare the solid red circles and the solid red squares in Fig. 3(a)). In contrast, macroscopic predictions (ref. 42 and eqn (2) for *θ*_dry_) suggest that the larger parallelepipeds should be more favorable for drying, because of the presence of four sharp corners at the bottom, which tend to destabilize the bubbles. This crossover is reflected also in the predictions for the drying contact angle in Fig. 2 (see the intersection of the green and the red lines therein).

**Fig. 4:**
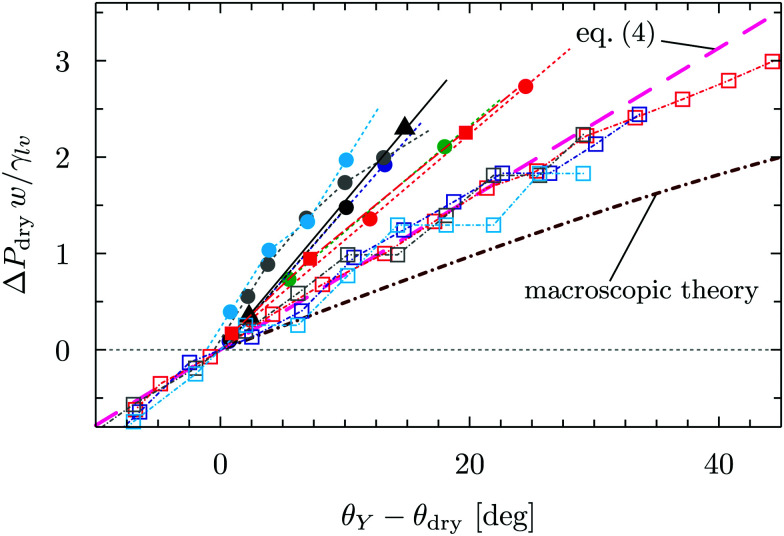
Dimensionless drying pressure as a function of the deviation of the substrate contact angle *θ*_Y_ from the drying angle *θ*_dry_ in Fig. 2. The cases considered in Fig. 3 are reported here with the same color code as in Fig. 3; the brown line stems from the dimensionless macroscopic theory for the groove and the magenta line is the linear fit of the groove data *via* (the dimensionless version of) eqn (4).

By using *σ* = 0.315 nm and *k*_B_*T* = 4.16 × 10^−21^ J it is possible to translate the results in Fig. 3 into experimentally relevant units. It is seen that the values of Δ*P*_dry_ can be larger than 20 MPa for the smallest three-dimensional pits (cylindrical and parallelepipedic with *w* = 1.9 nm and *θ*_Y_ ≥ 128°). As better discussed in Fig. 4, the drying pressure decreases with the size as Δ*P*_dry_ ∼ 1/*w*; for the cylindrical pit with *w* = 6.3 nm and *θ*_Y_ = 128°, this leads to Δ*P*_dry_ ≈ 2 MPa. The degree of confinement also plays an important role: for approximately the same dimensions (reported in the same colors in Fig. 3(a)) and contact angles (*θ* = 130°), the two-dimensional grooves are less likely to dry, with Δ*P*_dry_ < 10 MPa and <1 MPa for *w* = 1.9 nm and *w* = 6.6 nm, respectively.

Fig. 3(a) also highlights another surprising nanoscale effect, which permits the drying transition to occur for grooves at *θ*_Y_ < *θ*_wd_ = 135°. This result cannot be explained by the macroscopic theory according to which there is no drying possible for *θ*_Y_ < *θ*_wd_. In Fig. 3(b) the density distribution obtained by DFT shows that this corresponding destabilization of the liquid phase is related to the formation of nanoscale bubbles in the lower corners of the cavities which, however, are not compatible with the classical capillarity approximation for *θ*_Y_ < *θ*_wd_. In particular, in nanoscale confinement the formation of such minuscule bubbles is promoted by the long-ranged interaction of the fluid particles with the walls; the cause also includes the density oscillations (layering) induced by the various walls meeting at the wedges or corners. These various layers strongly interfere in the vicinity of wedges or corners, leading to specific distributions of the fluid density there (see Fig. 3(b) and (d)). As a result, the nanoscale bubbles in the corners significantly differ from the circular segments predicted by classical capillarity. However, for larger contact angles, the shape of the corner bubbles is similar to that of the macroscopic bubbles (Fig. 3(c)).

In order to rationalize the nanoscale results, we translate the pressure data in Fig. 3(a) into dimensionless quantities by multiplying them by the characteristic dimension *w* of the cavity and dividing them by *γ*_lv_. Fig. 4 shows that, if the drying pressures are plotted not as a function of *θ*_Y_ but as a function of the deviation of *θ*_Y_ from the geometry- and size-dependent drying contact angle *θ*_dry_ at coexistence, the dimensionless drying pressures approximately collapse on master curves *w*/*γ*_lv_Δ*P*_dry_ = *f*(*θ*_Y_ − *θ*_dry_), which depend on the geometry of the confinement. In particular, the groove results remarkably fall upon a single curve which, however, does not coincide with the macroscopic expectation (brown line in Fig. 4). It is expected that, for grooves with *w* ≫ *σ*, the master curve obtained from the DFT results should approach the master curve of the macroscopic theory. The rescaled plot in Fig. 4 summarizes graphically the two nanoscale corrections to the macroscopic models discussed above, both of which facilitate drying. The first one is the reduction of the angle *θ*_dry_ (Fig. 2). A second correction becomes apparent upon comparing within Fig. 4 the master curve obtained from DFT for the groove geometry with the corresponding curve derived from the macroscopic theory. It can be seen that the dependence of Δ*P*_dry_ on the chemistry (*θ*_Y_) of the substrate is magnified due to nanoscale corrections, as if the confinement was effectively enhanced. Fig. 4 also suggests that these nanoscale corrections can be effectively cast into the following empirical expression:4
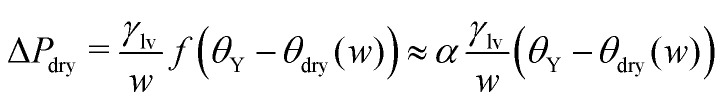
where a linear approximation has been introduced for *f*, and *θ*_dry_(*w*) has to be taken from the data in Fig. 2. This empirical formula is particularly appealing because it allows one to decouple the contribution to the drying pressure due to the cavity size, encoded by *w*, and due to its surface chemistry, encoded by *θ*_Y_. For the case of grooves the best fit is obtained for *α* = 0.078, which can be compared to the best linear fit to the macroscopic theory *α*_macro_ = 0.046 (magenta and brown lines in Fig. 4, respectively). This discrepancy is to be sought in the nanoscale effects discussed above and summarized in the conclusions, which are neglected in the macroscopic theory: finite widths of the interfaces, density oscillations at the walls, and increased hydrophobicity in confinement. Only for much larger widths of the structures, which are too large to be investigated directly *via* DFT, one would expect that these details become less relevant and that the macroscopic curve is gradually approached. The results for the pits scatter more broadly, with the data for the wider pits almost falling on a single curve, while the data for the very narrow pits clearly falling off, conceivably due to additional scale-dependent confinement effects. Unfortunately, neither for cylindrical nor for parallelepipedic pits an analytical macroscopic theory is available for comparison.

Summarizing this section, microscopic DFT calculations have shown that the classical capillarity theory for drying usually underestimates the drying pressure: nanoscale effects promote drying in hydrophobic confinement beyond the macroscopic expectations. Certain general features of the molecular interactions of the fluid with the walls, not accounted for in a macroscopic theory, trigger the formation of small regions with reduced density at the cavity corners, which allow drying at contact angles much smaller than macroscopically expected. Additionally, at a fixed contact angle, the drying pressure Δ*P*_dry_ in nanoscale confinement is increased as compared to the macroscopic one, thus extending the range of pressures within which perpetual superhydrophobicity is possible. Such nanoscale contributions can be quantified by the effective empirical drying model proposed in eqn (4).

## 4 Drying at hierarchical surfaces

Having analyzed macroscopic predictions and a microscopic theory of wetting, here we address drying on surfaces with a hierarchy of textures at the nanoscale and at larger scales which are amenable to a macroscopic description. The concept was first proposed in ref. 4 as a means to achieve perpetual superhydrophobicity on surfaces with micron-sized and larger textures. The rationale is that, under the conditions given below, the larger scale can inherit the self-recovery characteristics of the nanoscale coating, while preserving the technological advantages of having large textures. Indeed, previous studies have shown that hierarchical surfaces are capable of achieving very large apparent contact angles, low contact angle hysteresis, and superior mechanical robustness as compared to surfaces with only one tier of roughness.^28,56–61^ However, to the best of our knowledge, the experimental and conceptual difficulties exposed below have prevented heretofore the direct observation of perpetual superhydrophobicity being enabled by hierarchical surfaces. Here we briefly recapitulate the conditions under which a hierarchical surface structure can achieve the perpetual superhydrophobic state and discuss how these prescriptions can be realized and verified experimentally.

**Fig. 5:**
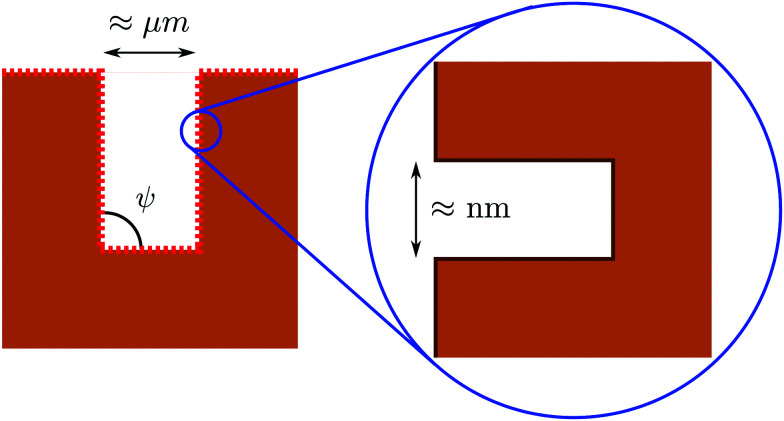
Illustration of a hierarchical surface with a tier of roughness at the nanoscale, which covers a second one at the micron (or even somewhat larger) scale (from ref. 31). The size and hydrophobicity of these textures can be designed in order to achieve perpetual superhydrophobicity at both scales; see the main text for further details.

A hierarchical surface with a structure similar to that shown in Fig. 5 can be made perpetually hydrophobic by combining the macroscopic and microscopic drying criteria uncovered in the previous sections:^4^

(1) Adopting a nanoscale coating or roughness which is perpetually superhydrophobic (Fig. 3);

(2) Controlling the solid fraction of the nanoscale coating such that the effective contact angle of the coated surface is compatible with the macroscopic drying condition (Fig. 1).

Only by satisfying both conditions, it can be ensured that the Wenzel state is eliminated both at the micro- and at the nanoscale and that, even if the transition to the Wenzel state happens to occur due to, *e.g.*, certain unfavorable external factors, the Cassie–Baxter state can be recovered by lowering the pressure to ambient values.

Since actual surfaces can feature roughness at various scales, it is useful to discuss how to realize and characterize their recovery properties based on the above criteria. One experimental difficulty is to verify that such criteria are satisfied both at the nanoscale and at the larger one, because their intrusion pressures are usually very different. For instance, using the Kelvin–Laplace law Δ*P*_KL_ = −2*γ*_lv_ cos *θ*_Y_/*w* for a groove of 10 µm, the typical intrusion pressure is estimated to be of the order of kPa, while for a size of 10 nm it is of the order of MPa. Such a separation between the intrusion pressures ensures that, under the usual operating conditions of the surface defined by the largest textures, one is sufficiently far from nanoscale intrusion. This feature is valid only for surfaces with a clear separation of scales and not for random surfaces for which a continuum of roughness dimensions is present. Usually experiments,^28,29,58^ aimed at demonstrating drying at hierarchical surfaces, test the surface by applying small pressures, for instance by the sessile drop method. At pressures of few tens of kPa it is only possible to demonstrate that the larger of the scales is capable of self-recovery. However, in order to demonstrate the occurrence of “monostability” or perpetual superhydrophobicity, it is necessary to explore much higher pressures, in order to induce liquid intrusion into the nanoscale coating and subsequently to observe its self-recovery upon pressure release. The consequences of having a non-perpetual nanoscale layer are of some technological importance and are well illustrated by ref. 59. In those experiments, liquid intrusion was achieved by changing the wettability of the liquid–surface pair by varying the composition of an ethanol–water mixture instead of directly changing the liquid pressure; this experiment demonstrated that, if surfaces with this nanoscale roughness are wet, recovery of the suspended state is impossible at both scales, rendering the Cassie–Baxter–Wenzel transition effectively irreversible with the usual limitations of non-perpetual superhydrophobicity. To conclude, there is a twofold open experimental challenge: fabricating well-characterized nanoscale textures capable of self-recovery and measuring drying conditions.

## 5 Conclusions

In this study we have elucidated the mechanism of spontaneous drying for liquids confined in hydrophobic cavities of varying size, geometry, and surface chemistry. Comparing the classical capillarity model with microscopic density functional theory has provided a route to quantify nanoscale effects, which facilitate drying in hydrophobic confinement (eqn (4)), by allowing drying in much broader ranges of contact angles and pressures.

The general mechanism for spontaneous drying of hydrophobic nano-cavities is related to the one operative at the macroscale. However, nanoscale effects lead to substantial qualitative and quantitative modifications. One such effect is the shift of the drying contact angles (*i.e.*, contact angles above which drying occurs) in nano-cavities to much lower values as compared to the macroscopic expectations. The macroscopic drying contact angles depend on the geometry but not on the size of the surface features; macroscopic values for right wedges or corners, *θ*_wd_ = 135° and *θ*_cd_ = 125.3°, are higher than the maximum contact angles typically achievable for water on flat surfaces. Nanoscale effects, however, shift the drying contact angles to much lower values. These shifts depend not only on the geometry but also on the size of the surface features. The shifts become more pronounced for smaller feature sizes. Moreover, the pressures up to which spontaneous drying occurs are increased very much due to nanoscale effects. These nanoscale effects, *inter alia*, originate from the long range of the fluid–wall interaction and from the oscillations of the liquid density induced by the repulsive part of the fluid–wall interaction together with the strong repulsion between fluid particles at short distances.

The present calculations have also revealed that the geometry of the confinement has a crucial impact on drying: the presence of extended hydrophobic walls and acute corners further facilitates drying, *e.g.*, by favoring it for cylindrical pores as compared to grooves. In general, these findings suggest that, in order to obtain perpetual superhydrophobicity, it is convenient to realize nanoscale hydrophobic cavities, which are capable of remaining permanently dry over a broad range of pressures, with levels of hydrophobicity attainable (for water) by using standard wall coatings (*θ*_Y_ < 110°). On the other hand, macroscopic cavities with sizes larger than a micrometer are advantageous in order to enhance certain superhydrophobic properties, such as self-cleaning and drag reduction; however, self-recovery is impossible for surfaces with average contact angles (*i.e.*, *θ*_Y_ ≈ 110°). With this understanding, hierarchical surfaces, consisting of micron-sized textures covered by a rough nanoscale coating, emerge as a practical route to achieve perpetual superhydrophobicity on texture sizes of technological relevance. In order to be effective, this strategy requires that the nanoscale cavities are capable of self-recovery and result in an effective contact angle larger than the macroscopic drying angle.

The coherent picture of drying across the scales provided by the present analysis not only offers useful design guidelines for perpetual superhydrophobicity, but also helps to understand the complex interplay of size, geometry, and degree of hydrophobicity which plays a crucial role in other technological (lyophobic porous materials) and biological (interactions of hydrophobic proteins) contexts. Finally, the present results are expected to encourage new experiments capable of realizing controlled nanoscale cavities and probing their drying behavior. This largely unexplored phenomenology is expected to be rich.

## Conflicts of interest

There are no conflicts to declare.

## Supplementary Material

SM-015-C9SM01049A-s001

## Appendices

### A Appendix: classical density functional theory

In this study we employ classical density functional theory in the version introduced by Rosenfeld in which fundamental measure theory is used to provide an expression for the excess free energy corresponding to the hard core repulsion among fluid particles.^52–54^ The attractive part of the fluid–fluid pair potential is taken into account within mean field theory and is of the van der Waals type with a cutoff at a distance 2.5*σ*. Further details concerning the theory and the numerical implementation are presented in ref. 55.

In the case of the rectangular grooves, the fluid–wall interactions are accounted for *via* an external wall potential *V*(***r***) = *V*_rep_(***r***) + *V*_att_(***r***), which is the sum of a repulsive and an attractive contribution. *V*_att_(***r***) is a linear superposition of the attractive part of the Lennard-Jones potential between the fluid and the wall particles.^4^*V*_rep_(***r***) is treated as a hard repulsion, leading to a depletion layer of thickness *σ*/2 where *σ* is the diameter of the fluid particles.

In the case of the pits, the substrate is built from atoms which are placed at the vertices of a simple cubic lattice, defined by the lattice constant 2*R*_w_, *i.e.*, twice the radius of the wall particles. The pair potential between fluid and wall particles is modeled *via* the Lennard-Jones potential:

5

where the summation runs over all positions **r**_*l*_ of the wall particles. The parameter *ε*_fw_ is the strength of the solid–fluid interaction and *σ*_fw_ = *R* + *R*_w_, where *R* = *σ*/2 is the radius of the liquid particles and *R*_w_ is the radius of the wall particles. A pit is created by removing all wall particles within the desired pit volume. We have chosen a ratio *R*_w_/*R* = 0.2 in order to generate smooth surfaces with weak corrugation.

We have checked that the details of how the wall–fluid interaction is modeled (*e.g.*, Lennard-Jones repulsion *versus* hard-core repulsion) are of very little importance for the extrusion behavior when the results are compared for two fluid–wall interactions characterized, however, by the same Young contact angle. More specifically, we have repeated the calculations for the groove geometry by using the Lennard-Jones pair potential between fluid and wall particles as described above, instead of the hard-core repulsion used in the main text. The corresponding result confirmed that the extrusion/intrusion behavior of a groove with a nominal width of 5.9*σ* essentially maps onto that of a 6*σ* wide groove with hard repulsive walls.

### B Appendix: determination of the drying pressure Δ*P*_dry_

The minimization procedure, which is used in DFT for determining the equilibrium fluid number densities, is always initialized in a configuration in which the grooves or pits are completely filled with liquid (Wenzel state). The DFT calculations are performed at various pressures, starting at a small value and increasing the pressure in small steps. For values of Δ*P* smaller than Δ*P*_dry_ the Wenzel state is unstable and the numerical iteration converges to the Cassie–Baxter state (pit filled with vapor). Δ*P* is subsequently increased up to a value at which the Wenzel state remains stable (or metastable). We have chosen Δ*P*_dry_ as the center of the pressure interval between the highest pressure at which the Wenzel state is still unstable and the lowest pressure at and above which a stable Wenzel state is found.

## References

[cit1] Barthlott W., Neinhuis C. (1997). Planta.

[cit2] Scardino A., Zhang H., Cookson D., Lamb R., de Nys R. (2009). Biofouling.

[cit3] Rothstein J. P. (2010). Annu. Rev. Fluid Mech..

[cit4] Giacomello A., Schimmele L., Dietrich S., Tasinkevych M. (2016). Soft Matter.

[cit5] Dietrich S. , in Phase Transitions and Critical Phenomena, ed. C. Domb and J. L. Lebowitz, Academic Press, 1988, ch. 1, vol. 12, pp. 1–218

[cit6] Berne B. J., Weeks J. D., Zhou R. (2009). Annu. Rev. Phys. Chem..

[cit7] Giovambattista N., Lopez C. F., Rossky P. J., Debenedetti P. G. (2008). Proc. Natl. Acad. Sci. U. S. A..

[cit8] Huang D. M., Chandler D. (2000). Proc. Natl. Acad. Sci. U. S. A..

[cit9] Miller T. F., Vanden-Eijnden E., Chandler D. (2007). Proc. Natl. Acad. Sci. U. S. A..

[cit10] Mondal J., Morrone J. A., Berne B. (2013). Proc. Natl. Acad. Sci. U. S. A..

[cit11] Chandler D. (2005). Nature.

[cit12] Roth R., Gillespie D., Nonner W., Eisenberg R. E. (2008). Biophys. J..

[cit13] Aryal P., Sansom M. S., Tucker S. J. (2015). J. Mol. Biol..

[cit14] Checco A., Rahman A., Black C. T. (2014). Adv. Mater..

[cit15] Patankar N. A. (2004). Langmuir.

[cit16] Savoy E. S., Escobedo F. A. (2012). Langmuir.

[cit17] Giacomello A., Chinappi M., Meloni S., Casciola C. M. (2012). Phys. Rev. Lett..

[cit18] Krupenkin T. N., Taylor J. A., Wang E. N., Kolodner P., Hodes M., Salamon T. R. (2007). Langmuir.

[cit19] Vrancken R. J., Kusumaatmaja H., Hermans K., Prenen A. M., Pierre-Louis O., Bastiaansen C. W., Broer D. J. (2009). Langmuir.

[cit20] Manukyan G., Oh J., Van Den Ende D., Lammertink R., Mugele F. (2011). Phys. Rev. Lett..

[cit21] Kavousanakis M. E., Chamakos N. T., Ellinas K., Tserepi A., Gogolides E., Papathanasiou A. G. (2018). Langmuir.

[cit22] Papathanasiou A. G. (2018). Curr. Opin. Colloid Interface Sci..

[cit23] Cheng Z., Lai H., Zhang N., Sun K., Jiang L. (2012). J. Phys. Chem. C.

[cit24] Feng X., Feng L., Jin M., Zhai J., Jiang L., Zhu D. (2004). J. Am. Chem. Soc..

[cit25] Caputo G., Cortese B., Nobile C., Salerno M., Cingolani R., Gigli G., Cozzoli P. D., Athanassiou A. (2009). Adv. Funct. Mater..

[cit26] Sun T., Wang G., Feng L., Liu B., Ma Y., Jiang L., Zhu D. (2004). Angew. Chem., Int. Ed..

[cit27] Liu G., Fu L., Rode A. V., Craig V. S. (2011). Langmuir.

[cit28] Verho T., Korhonen J. T., Sainiemi L., Jokinen V., Bower C., Franze K., Franssila S., Andrew P., Ikkala O., Ras R. H. (2012). Proc. Natl. Acad. Sci. U. S. A..

[cit29] Li Y., Quéré D., Lv C., Zheng Q. (2017). Proc. Natl. Acad. Sci. U. S. A..

[cit30] Checco A., Ocko B. M., Rahman A., Black C. T., Tasinkevych M., Giacomello A., Dietrich S. (2014). Phys. Rev. Lett..

[cit31] Giacomello A., Schimmele L., Dietrich S. (2016). Proc. Natl. Acad. Sci. U. S. A..

[cit32] Giacomello A., Chinappi M., Meloni S., Casciola C. M. (2013). Langmuir.

[cit33] Xiang Y., Huang S., Lv P., Xue Y., Su Q., Duan H. (2017). Phys. Rev. Lett..

[cit34] Lefevre B., Saugey A., Barrat J.-L., Bocquet L., Charlaix E., Gobin P.-F., Vigier G. (2004). J. Chem. Phys..

[cit35] Guillemot L., Biben T., Galarneau A., Vigier G., Charlaix É. (2012). Proc. Natl. Acad. Sci. U. S. A..

[cit36] Grosu Y., Li M., Peng Y.-L., Luo D., Li D., Faik A., Nedelec J.-M., Grolier J.-P. (2016). ChemPhysChem.

[cit37] Tinti A., Giacomello A., Grosu Y., Casciola C. M. (2017). Proc. Natl. Acad. Sci. U. S. A..

[cit38] Leung K., Luzar A., Bratko D. (2003). Phys. Rev. Lett..

[cit39] Luzar A. (2004). J. Phys. Chem. B.

[cit40] Sharma S., Debenedetti P. G. (2012). J. Phys. Chem. B.

[cit41] Prakash S., Xi E., Patel A. J. (2016). Proc. Natl. Acad. Sci. U. S. A..

[cit42] Lisi E., Amabili M., Meloni S., Giacomello A., Casciola C. M. (2018). ACS Nano.

[cit43] Rejmer K., Dietrich S., Napiórkowski M. (1999). Phys. Rev. E: Stat. Phys., Plasmas, Fluids, Relat. Interdiscip. Top..

[cit44] Roth R., Parry A. O. (2011). Mol. Phys..

[cit45] Roth R., Parry A. O. (2012). J. Phys. Soc. Jpn..

[cit46] Malijevský A., Parry A. O. (2015). Phys. Rev. E: Stat., Nonlinear, Soft Matter Phys..

[cit47] Hauge E. H. (1992). Phys. Rev. A: At., Mol., Opt. Phys..

[cit48] Evans R., Marconi U. M. B., Tarazona P. (1986). J. Chem. Phys..

[cit49] Lum K., Luzar A. (1997). Phys. Rev. E: Stat. Phys., Plasmas, Fluids, Relat. Interdiscip. Top..

[cit50] Meloni S., Giacomello A., Casciola C. M. (2016). J. Chem. Phys..

[cit51] Amabili M., Giacomello A., Meloni S., Casciola C. M. (2016). J. Phys.: Condens. Matter.

[cit52] Rosenfeld Y. (1989). Phys. Rev. Lett..

[cit53] Rosenfeld Y., Schmidt M., Löwen H., Tarazona P. (1997). Phys. Rev. E: Stat. Phys., Plasmas, Fluids, Relat. Interdiscip. Top..

[cit54] Roth R. (2010). J. Phys.: Condens. Matter.

[cit55] Singh S. L., Schimmele L., Dietrich S. (2015). Phys. Rev. E: Stat., Nonlinear, Soft Matter Phys..

[cit56] Li W., Amirfazli A. (2008). Soft Matter.

[cit57] Bhushan B., Jung Y. C., Koch K. (2009). Philos. Trans. R. Soc., A.

[cit58] Kwon Y., Patankar N., Choi J., Lee J. (2009). Langmuir.

[cit59] Boreyko J. B., Baker C. H., Poley C. R., Chen C.-H. (2011). Langmuir.

[cit60] Bae W.-G., Kim H. N., Kim D., Park S.-H., Jeong H. E., Suh K.-Y. (2014). Adv. Mater..

[cit61] Ghosh M., Rao G. M. (2019). Phys. Rev. Appl..

