# Supplementary material for: Recovering superhydrophobicity in nanoscale and macroscale surface textures
Source: Soft Matter. 2019 Aug 20;15(37):7462–71. doi: 10.1039/c9sm01049a (PMC8751625; doi:10.1039/c9sm01049a)
Supplement: SM-015-C9SM01049A-s001 [file SM-015-C9SM01049A-s001.pdf]

Electronic Supplementary Material (ESI) for Soft Matter.
This journal is © The Royal Society of Chemistry 2019

Electronic Supplementary Information Recovering superhydrophobicity in nanoscale and macroscale surface textures

Alberto Giacomello, Lothar Schimmele, Siegfried Dietrich, Mykola Tasinkevych

1 Characterization of the wall textures

Here we provide all parameters which characterize the wall textures studied in the DFT calculations, i.e., grooves, cylindrical, parallelepipedic, and inkbottle pits. The height of the grooves and of all pits is $20\,\sigma$. Quasi-twodimensional grooves with widths of $6\,\sigma$, $11\,\sigma$, $16\,\sigma$, and $21\,\sigma$ have been used. Right circular cylinders with diameters of $6\,\sigma$, $8\,\sigma$, $10\,\sigma$, $12\,\sigma$, $15\,\sigma$, and $20\,\sigma$ have been analyzed. The "inkbottle" pit is actually a right circular truncated cone with upper diameter (cavity mouth) of $6\,\sigma$, with lower diameter (pit bottom) of $12\,\sigma$, and with height $20\,\sigma$. The parallelepipedic pits have square cross sections with vertical sidewalls; sides of $6\,\sigma$, $8\,\sigma$, $10\,\sigma$, $12\,\sigma$, $15\,\sigma$, and $20\,\sigma$ have been used in the calculations of $\theta_{\rm dry}$ (Fig. 2 in the main text); for the determination of $\Delta P_{\rm dry}$ (Figs. 3 and 4 in the main text), due to the higher computational cost, only a side of $6\,\sigma$ has been used. All pits have weakly corrugated walls, due to the aforementioned procedure of building the wall from small wall particles on a lattice.

2 Supplementary figures

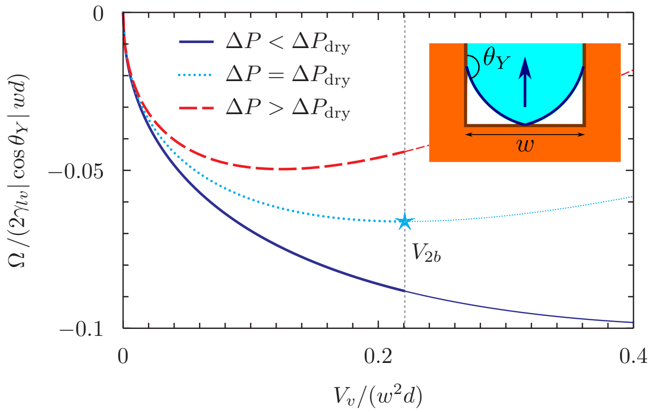

Figure S1: Profile of the grand canonical potential Ω for a macroscopic, infinitely deep groove as function of the bubble volume V_v (white area in the inset); the system is taken to be translationally invariant in the direction normal to the plane of the figure. The bubble volume is measured in units of $w^2 d$ where w is the groove width and d is its length in the normal direction. The grand canonical potential is measured in units of wd, the liquid-vapor surface tension γ_{lv}, and $\cos\theta_Y$. The profile is obtained by confined classical nucleation theory [1] assuming that two identical bubbles form in the two bottom corners; such bubbles have variable curvature and meet the groove walls with a contact angle equal to $\theta_Y = 145°$. The blue area indicates the liquid. For each V_v, the grand canonical potential is computed via Eq. [3] of the main text. The vertical line at $V_v = V_{2b}$ indicates the volume at which the two bubbles touch each other at the center of the groove, as shown graphically in the inset. The three lines correspond to Ω for three pressures; in units of the Kelvin-Laplace pressure $\Delta P_{\text{KL}} = -2\gamma_{lv}\cos\theta_Y/w$, $\Delta P = 0.2\,\Delta P_{\text{KL}}$ (blue full line), $\Delta P = \Delta P_{\text{dry}} = 0.3\,\Delta P_{\text{KL}}$ (light blue dotted line), and $\Delta P = 0.4\,\Delta P_{\text{KL}}$ (red dashed line). Following the vertical arrow, merging amounts to shifting the dark blue liquid-vapor interface upwards. The drying pressure ΔP_{dry} is determined by the minimum ($\star$) of Ω occurring at $V_v = V_{2b}$ (light blue dotted line): for all $\Delta P > \Delta P_{\text{dry}}$ there is no drying (red dashed line) because the equilibrium value $(V_v)_{eq}$ of V_v is at a volume for which the two bubbles are too small to touch each other. For all $\Delta P < \Delta P_{\text{dry}}$ the groove is dry because $(V_v)_{eq}$ would grow to V_{2b} and beyond (blue full line, see also Fig. S2). The thin lines for $V_v > V_{2b}$ are the continuation of Ω in the hypothetical case that the two bubbles could grow indefinitely without touching each other (two independent wedges). Actually, in a finite groove, the curve of Ω versus V_v jumps to the dash-dotted curve shown in Fig. S2, which represents configurations detached from the bottom wall of the groove.

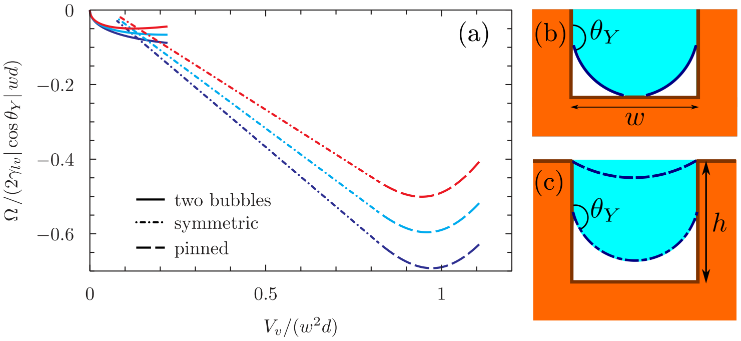

Figure S2: (a) Profile of the grand canonical potential Ω for a macroscopic groove of finite height h, in a broader range of V_v than the one shown in Fig. S1. The same pressures, values of θ_Y, units, and color code of Fig. S1 are used. (b)-(c) The sketches show the three bubble morphologies which minimize the grand canonical potential for different values of V_v: after the two bubbles ((b), solid lines) merge, a meniscus with the shape of a circular arc, meeting the vertical walls with contact angle θ_Y, forms ((c), dash-dotted lines), which eventually is pinned at the groove mouth ((c), dashed lines) [1].

References

[1] A. Giacomello, M. Chinappi, S. Meloni and C. M. Casciola, *Phys. Rev. Lett.*, 2012, **109**, 226102.
